# National Mesothelioma Virtual Bank: A standard based biospecimen and clinical data resource to enhance translational research

**DOI:** 10.1186/1471-2407-8-236

**Published:** 2008-08-13

**Authors:** Waqas Amin, Anil V Parwani, Linda Schmandt, Sambit K Mohanty, Ghada Farhat, Andrew K Pople, Sharon B Winters, Nancy B Whelan, Althea M Schneider, John T Milnes, Federico A Valdivieso, Michael Feldman, Harvey I Pass, Rajiv Dhir, Jonathan Melamed, Michael J Becich

**Affiliations:** 1 Departments of Biomedical Informatics, University of Pittsburgh, PA, USA; 2 Department of Pathology, University of Pittsburgh School of Medicine, PA, USA; 3 Department of Epidemiology, University of Pittsburgh School of Medicine, Pittsburgh, PA, USA; 4 Department of Pathology, University of Pennsylvania School of Medicine, Philadelphia, PA, USA; 5 Department of Pathology, New York University School of Medicine, New York, NY, USA; 6 Department of Cardiothoracic Surgery, Division of Thoracic Surgery and Thoracic Oncology, New York University School of Medicine, New York, NY, USA

## Abstract

**Background:**

Advances in translational research have led to the need for well characterized biospecimens for research. The National Mesothelioma Virtual Bank is an initiative which collects annotated datasets relevant to human mesothelioma to develop an enterprising biospecimen resource to fulfill researchers' need.

**Methods:**

The National Mesothelioma Virtual Bank architecture is based on three major components: (a) common data elements (based on College of American Pathologists protocol and National North American Association of Central Cancer Registries standards), (b) clinical and epidemiologic data annotation, and (c) data query tools. These tools work interoperably to standardize the entire process of annotation. The National Mesothelioma Virtual Bank tool is based upon the caTISSUE Clinical Annotation Engine, developed by the University of Pittsburgh in cooperation with the Cancer Biomedical Informatics Grid™ (caBIG™, see ). This application provides a web-based system for annotating, importing and searching mesothelioma cases. The underlying information model is constructed utilizing Unified Modeling Language class diagrams, hierarchical relationships and Enterprise Architect software.

**Result:**

The database provides researchers real-time access to richly annotated specimens and integral information related to mesothelioma. The data disclosed is tightly regulated depending upon users' authorization and depending on the participating institute that is amenable to the local Institutional Review Board and regulation committee reviews.

**Conclusion:**

The National Mesothelioma Virtual Bank currently has over 600 annotated cases available for researchers that include paraffin embedded tissues, tissue microarrays, serum and genomic DNA. The National Mesothelioma Virtual Bank is a virtual biospecimen registry with robust translational biomedical informatics support to facilitate basic science, clinical, and translational research. Furthermore, it protects patient privacy by disclosing only de-identified datasets to assure that biospecimens can be made accessible to researchers.

## Background

The latest advancements in translational medicine directed the path towards highly demanded biomarker validation studies that focused on the identification of new cancer biomarkers for cancer research community. These studies lead toward the development of biobanks that are capable of providing well annotated high quality tissue samples that can meet the current demands of cancer research community. This objective is achieved successfully by employing a well designed informatics architecture that balances and supports the routine activities of a biobank [1]. Tissue Banking Informatics is relatively novel area of biomedical informatics that deals with collecting and storage of biospecimens with associated clinicopathology annotation. The importance of the development of tissue banks has been realized for the last decade with the advent of advanced computer technology that is utilized in the development of tissue bank informatics infrastructure. These systems have the capability to include the managing aspects of biobank that can function as central role in the development of translational research initiatives [2-6].

National Mesothelioma Virtual Bank (NMVB), funded by The Centers for Disease Control and Prevention (CDC) in association with National Institutes of Occupational Health and Safety (NIOSH), is an example of an exceptional and progressive tissue banking effort. The main objective of this resource is to provide mesothelioma pleural, pericardial and peritoneal tissue samples along with blood and DNA samples with associated cancer registry, clinicopathology, recurrence and therapy related data on a web based interface to meet the growing need of cancer research community. The multimodal data is integrated from various hospital systems into a web-based architecture which is built upon a set of common data elements (CDEs) that provide semantic and syntactic interoperability across multiple institutions to facilitate translation research. During the development of this resource we applied the lessons learnt from the earlier collaborative projects: fundamental components and standards were employed from Cooperative Breast Cancer Tissue Resource (CBCTR), Pennsylvania Cancer Alliance Bioinformatics Consortium (PCABC), Cooperative Prostate Cancer Tissue Resource (CPCTR), and the cancer Data Standard Repository (caDSR) model of caBIG [6-11].

The manuscript highlights the infrastructure components that were used to build this virtual biobank allowing it to collect clinically annotated tissues from various clinical settings which meet with statutory and best practice guidelines. This resource provides a web-based publicly accessible database that organizes the data by CDE standards and allows the end users to query on de-identified information within the warehouse through a controlled "point and click" interface.

The resource also allows web-based requests for tissues, provides a fair review process, delivers tissues to scientists, permits growth and continual updates and integration with other ongoing efforts, and markets the whole process. Here we describe all the components that make up this comprehensive NMVB informatics architecture, discuss the use of standards, and outline the previous collaborative biorepository experiences of the team at the University of Pittsburgh, Department of Biomedical Informatics [12].

## Methods

### Collaborative Institutions

The resource currently has three participating institutions: University of Pennsylvania (U. Penn), University of Pittsburgh (U. Pitt) and New York University (NYU). The other participants supporting this effort are the Mesothelioma Applied Research Foundation (MARF), University of Hawaii, University of Vermont, and Fox Chase Cancer Center. The collaborative efforts make this resource capable of providing diverse biospecimens including fresh frozen tissues, paraffin blocks, tissue microarrays (TMAs), blood and DNA samples with clinicopathologic and follow-up data from a wide variety of medical care settings spread across rural and urban regions.

### Inclusions/Exclusion Criteria of Cases into the Resource

#### Prospective Collection Criteria

The criteria for participation in the NMVB prospective study is that the individuals are receiving or seeking medical care for mesothelioma are 18 years old or older and can provide informed consent. This is usually obtained at either a doctor's office, clinic visits or upon hospital admission.

The only exclusion criteria are patients younger than 18 years old and prisoner-patients, who are excluded based on federal limitations. As very few patients are likely to not be eligible for inclusion based on these criteria, the racial, gender and ethnic characteristics of the individuals approached for participation in this NMVB Registry reflect the demographics of patients receiving or seeking medical care at the mesothelioma clinics at the member institutions of the consortium. No individuals are excluded from participation in the NMVB Registry on the basis of race, ethnicity, gender or HIV status.

#### Retrospective collection Criteria

The NMVB registry utilizes an honest broker system for identifying and enrolling eligible retrospective cases into the NMVB database. The honest broker system is also utilized by the Tissue bank with Institutional Review Board (IRB) approval for collecting excess tissue and biological materials. The retrieval of samples from archived residual biospecimens that have previously been removed for clinical treatment and pathology diagnosis as well as retrospective review of associated medical records for annotation purposes fall within the IRB's exempt category [13].

### Patient Health Information Protection and De-Identification Process

The NMVB has designed its procedures to protect the confidentiality and privacy of human subjects and IRB approval has been obtained for all activities. NMVB uses a decentralized system for specimen and data collection and storage. Each case is assigned a de-identified NMVB number. The only link to patient identity is held in reserve locally within the institution and there are no links directly connecting specimens or data to patients.

The NMVB database is available to the research community on a public web site but it has been designed to produce only de-identified datasets upon query. This is possible because only de-identified data sets exist which are compliant with the 'safe harbour' approach to HIPPA (Health Insurance Portability and Accountability Act) [14]. The "safe-harbor" approach entails removal of all 18 identifiers enumerated at section 164.514(b) [2] of the regulations. Thus for example, a participant's age is presented as age rage, rather than the date of birth, an approach which protects the identity while still providing sufficient information for research purposes. All data requests are tracked in the secure NMVB web-based data query tool regardless of whether the purpose is clinical or research related.

The Honest Broker acts as a barrier between fully identified confidential clinical patient information and the completely de-identified data made available to the research community. An honest broker is an individual, organization or system acting for or on behalf of the covered entity to collect and provide health information to the investigators in such a manner whereby it would not be reasonably possible for the investigators or others to identify the corresponding patients-subjects directly or indirectly. The honest broker cannot be one of the investigators or researcher. A researcher may use the services of an honest broker service to obtain the Protected Health Informations in a de-identified manner. De-identification means that the patient-subjects cannot be identified by researchers or others directly or indirectly through identifiers link to the patient-subject. This honest broker service will de-identify medical record information by automated or manual methods. All honest broker services are approved in advance by both the IRB of record and UPMC. If an honest broker service is not part of the UPMC covered entity, a valid business associate agreement with UPMC is executed with UPMC in order to access UPMC-held Protected Health Informations for de-identification. If an honest broker system/service is to be used to obtain de-identified Protected Health Informations, this fact must be identified in the study's IRB submission. The honest brokers are individuals who have clinical responsibilities as tissue bankers in the Health Sciences Tissue Bank (HSTB), postdoctoral fellows to manage the pathology data or as cancer registry specialists in the UPMC Network Cancer Registry. Based on their clinical job duties, their educational backgrounds and experiences vary. Depending on the nature of the projects, these bankers can work autonomously or collaboratively to meet biospecimen and/or data needs (please refer to additional file 1) [13].

### Development of the Common Data Elements

Common Data Elements were developed to allow annotation of cases and accomplish characterization of biospecimens collected from different collaborating sites. The development of CDEs for the resource has recently been described in detail in a related publication from our group [15]. In summary, the CDE development was by joint consensus of participants (including domain experts from various subcommittees) under the leadership supervision of the NMVB Coordinating Committee. The CDEs include cancer registry data (demographic, epidemiology and follow-up) at the patient level, pathology data at the specimen level including data elements to elaborate TNM staging and tumor grading, along with block level annotation and genotype data [16]. In this process the CDE sub-committee used the experiences gained from previous projects like the PCABC [8], CPCTR [9], caBIG [11], Early Detection Research Network (EDRN) [17] and Specialized Programs of Research Excellence (SPOREs) [18]. The major standards used to develop the CDEs include the NAACCR Data Standards for Cancer Registries [19], CAP Cancer Protocol and Checklist [20], the ADASP – Association of Directors of Anatomic and Surgical Pathology (ADASP) [21] and the American Joint Committee on Cancer (AJCC) Cancer Staging Manual [22]. The utilization of all the above mentioned standards facilitates the development of CDEs that provides an informatics support which is quite ample to achieve both syntactic and semantic interoperability across various systems [15].

### Data Collection and Method of Data Transmission

#### Retrospective Collection

Each collaborative site collects data and tissue samples. Once a case becomes available the pathologists at each site review the surgical pathology report and all histological sections of the tissue facilitating the selection of the mesothelioma case. Then the pathologist selects key slides, referred to as matrix slides, according to a standardized protocol with specific features of the case which is likely to be of interest for scientific investigators. Afterwards the detailed data elements are collected on these slides, and the slides are used to obtain the corresponding matrix blocks which represent the core specimen components of the Resource. This results in block-level annotation of the case with recording of important information about the case such as the size and type of tumor present in each block of interest. Overall, this process increases the utility of the resource many fold as the block level annotation leads to finding cases queried by specific criteria at a later point. This review up front also facilitates the retrieval of such blocks when a request is received at a later point. After the review of pathology data, the data managers and certified tumor registrars retrieve the clinical and follow-up data for cases enrolled in the study. The data are derived in part from the tumor registries of the various hospitals and institutions, which include the Anatomic Pathology Laboratory Information system (APLIS) (b) Cancer Registry Information System (CRIS), and (c) Tissue Banking Inventory System (TBIS).

#### Prospective Collection

To collect mesothelioma cases prospectively, patient are being consented at the physician office or hospitals at each collaborative site. At the time of patient enrollment in the study prospectively a NMVB Health Assessment Questionnaire is handed over to the patient. The purpose of this questionnaire is to collect patient demographic, epidemiology and past medical history data. Additionally, detailed clinical information is obtained by direct review of and extraction of information from patient charts, from consultation with outpatient referring physicians, and from direct patient interviews performed by cancer registrars and clinical nurses. Data is collected and annotated using common data entry paper forms that are correlated with the CDEs developed by the Resource [12]. Finally, collaborative sites transfer de-identified data to the central database either manually using the web-based data entry tool or electronically in spreadsheets.

### Quality Assurance and Check of NMVB Datasets

The transmitted data from collaborative sites is reviewed at the NMVB coordinating (database) site, which processes the data according to quality assurance (QA) and quality check (QC) protocols every four months that has been established by the resource. The process of QA/QC is performed to locate any missing datasets and errors performed during data recording. The value of each data set is matched with the CDE dictionary to make certain the standards are being followed during data recording. Only the standardized data is uploaded into the database at regular basis, and data with errors is sent back to the corresponding sites along with explanations of rejection. The correction of the data is responsibility of each collaborative site. To ensure the quality of data an audit review process has been established.

The resource selects 20% of the newly entered cases to be reevaluated by honest brokers, cancer registrars and data managers. After completion of their review, the audit reviewers submit a report of their findings and recommendations to the resource. The resource members discuss their findings in the next general meeting of the NMVB Coordinating Committee and make plans to implement the proposed recommendations [12].

The NMVB resource has periodic QA/QC of pathology data by assessing inter-observer concordance for the resource pathologists. These consist of two methods which include 1) the joint review of cases at meetings and 2) independent review of cases circulated among sites. For joint QA review during meetings, resource pathologists review up to 5 cases from each site, with emphasis on the 5 "matrix slides" selected by each pathologist at each site to include areas of difficulty or likely diagnostic differences. Joint review of cases is performed on a multi-headed microscope that permits pathologists to discuss diagnostic differences and set thresholds.

Independent review of cases is performed by individual pathologist at each site on cases sent from the other collaborative sites at regular intervals. The data mangers at each site will randomly select the cases for QA review from those added to the Resource within certain cut-off dates. The review will be documented by completing the "matrix" fields and select "histology" fields of the NMVB database. Any areas that show a high level of discrepancy will be determined by data mangers and communicated to resource pathologists. The Pathology Subcommittee will then discuss their findings in the subsequent general meeting of the Coordinating Committee and provide a report with recommendations as indicated by their findings [12].

### Mesothelioma Virtual Bank Central Data Warehouse

The NMVB database is based on an informatics model that aids in achieving semantic and syntectic interoperability by describing the common data elements in the form of metadata or data descriptors and by using a controlled vocabulary in order to make the data understandable and sharable for end-users. The system architecture is designed to provide query speed and high security as well as expansion capabilities for incorporating new data elements or integrating existing systems at participating institutions. Emphasis has been placed to provide user access at three levels (a) NMVB statistical data for public view (b) approved investigator database query that allows seeing individual patient de-identified clinical data and (c) data manger access to query and edit the stored data. Patient privacy is of utmost importance at all levels of user's access.

The NMVB data warehouse is constructed using information models captured as UML class diagrams. Unified Modeling Language (UML) is a non-proprietary language for constructing, visualizing, and documenting the artifacts of software engineering. The information model for CDEs was created to establish a structurally aligned generalized relationship [23]. Enterprise Architect (EA) software has been utilized to develop the UML class diagrams [24].

The NMVB web based query tool is based on the caTISSUE Clinical Annotation Engine (CAE) application. CAE was originally developed by the University of Pittsburgh as part of the National Cancer Institute's (NCI) Cancer Biomedical Informatics Grid (caBIG) program [25]. The NMVB database allows researches to search clinically annotated Mesothelioma biospecimens via a web interface in real time. The database is made available through a publicly available website [26]. The database warehouse facilitates standardized clinical annotation structure and incorporates variety of data sets from different data sources. Two methods of annotation have been adopted manually using web based data entry tool and data imported electronically through XML files [27].

### Marketing of Resource Specimens

Various types of media resources are being utilized for the advertisement of the NMVB biospecimens and services to the research community as follow:

**1**. *The NMVB web site *has been developed and maintained at Department of Biomedical Informatics at University of Pittsburgh [28]. The web site includes general information about the resource and different type of specimens available to the research community.

**2**. *Mass E- mailings to Investigator*: Announcement letters of invitation to utilize the resource are sent via e-mail to investigators that have published articles in tissue- based mesothelioma research. Additional names were provided by investigators who visited the NMVB booth at scientific meetings. A mechanism is provided for any email subscriber to "opt out" from the mailing list at the time any mass mailing is distributed.

**3**. *Advertisements *are placed in specialty scientific journals, research society newsletters, fliers at research meetings, and through free listings in journals and websites.

**4**. *Posters and podium presentations *regarding the practical use and the Resource specimens at research meetings.

**5**. *Marketing booths *at scientific research meetings, in collaboration to CDC and NIOSH or as individual standalone booths. Marketing surveys were conducted at scientific research meetings as well as with well-known mesothelioma researchers to estimate what resources the NMVB should focus on providing to the research investigators.

## Results

### NMVB Biospecimens Collection

The NMVB resources hold over 650 archived mesothelioma cases and prospective cases. The resource provides more than 775 biospecimes that are accrued from surgical resections and biopsies and also includes whole blood and DNA samples.

This collection is made possible by the collaborative efforts of University of Pennsylvania, University of Pittsburgh and New York University. At the end of second quarter of 2008 we project the resource to have over 700 annotated cases of pleural, peritoneal and pericardial mesothelioma specimens along with blood and DNA samples available to the mesothelioma research community. The majority of these cases consist of archival paraffin blocks from surgically treated patients. In addition the first Tissue Microarray (TMA) has been made available to investigators along with de-identified clinicopathology and follow-up data. IATA Dangerous Goods Regulations [29] are implemented for the special handling and safe shipping of biospecimen to investigators. All major courier companies acknowledge IATA measures of shipping biohazardous supplies by air.

**Figure 1:**
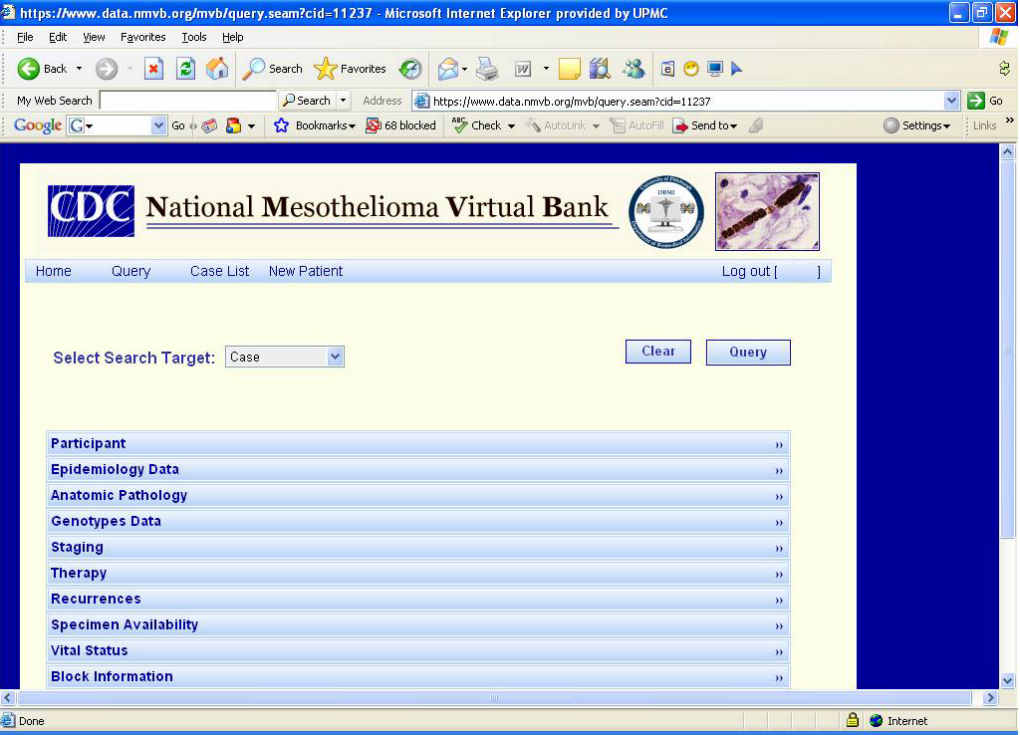
**Presents the NMVB approved investigator query tool.** This view provides de-identified data sets upon end-user query.

The NMVB common data elements (CDE) are developed on NAACCR Data Standards for Cancer Registries [19], CAP Cancer Protocol and Checklist [20], the ADASP [21] and the American Joint Committee on Cancer (AJCC) Cancer Staging Manual [22] standards to build mesothelioma CDE datasets. The NMVB web based Query Tool is based upon CAE version 2.0. The query tool is secure password protected and only investigator by approved IRB and Scientific Review Committee are capable to access the database. It permits end-users to develop their own case lists for their applications and quarry the data related to the specimen cases they have received from the resource for their approved studies. The query results shows only de-identified datasets associated with each approved case through disease and specimen pre-defined views of the data set (Figure 1).

**Figure 2:**
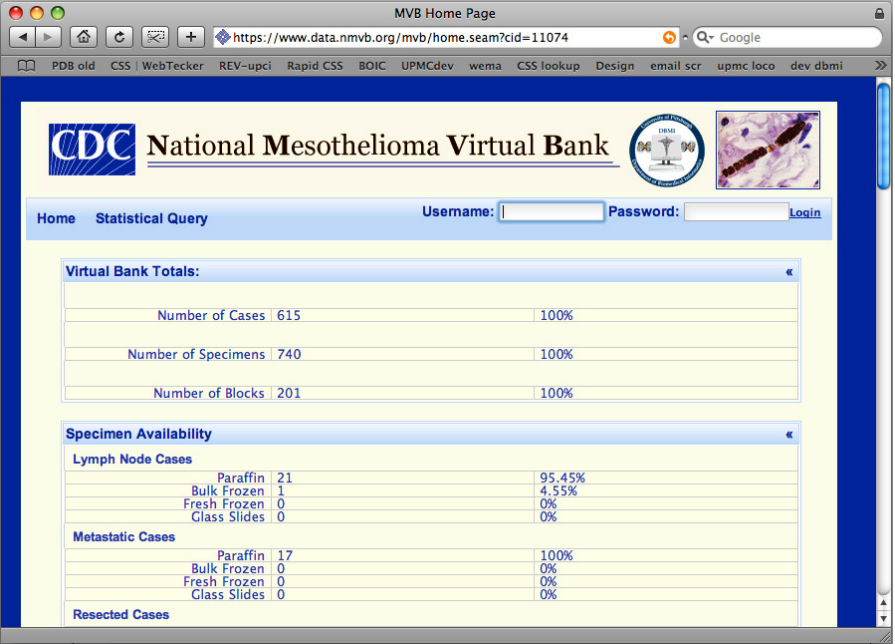
**Presents the NMVB statistical summary for public view.** This view provides overall statistics on all the cases and biospecimens stored into the resource.

The NMVB Statistical Database for Public View is open to the general public. The statistical data base for public query provides summary information on all the mesothelioma cases and their associated biospecimens stored into biorespoitory. The result page shows the number of cases, specimens and blocks in the database that match with query criteria of the investigator and general statistics on a limited number of data elements. By utilizing this public view database the investigator could be able to gain enough information to decide if the resource has sufficient biospecimens to fulfill his experimental requirements (Figure 2).

## Discussion

The growing importance of cross-institutional translational research and advancement in the development of informatics tools that are capable of analyzing clinical tissue sample has increased the need of research community for high quality and well annotated biospecimens. To fulfill research community's requirements the NMVB has established an integrated mesothelioma biobank and web base database query tool. This system is built on an underlying architect of common data elements for characterization of tissue samples and clinical follow-up data, supported by an essential quality assurance process. By the development and implementation of NMVB database, the resource developed a web based query tool to make investigators knowledgeable of the resource biospecimens. Finally, the NMVB resource has developed efforts to market the specimens, and a process for requesting samples and data by investigators that involves an independent research evaluation panel (REP) of professionals on mesothelioma, biostatistics, and pathology that are not part of resource.

### Clinical Annotation and development of federated Model NMVB datasets

The data mangers at each collaborative site are responsible for clinical annotation of biospecimen and collect related information manually from variety of data sources. The collected data is then integrated, de-identified and standardized according to approved study protocols. The integration is a process in which a selected patient data is collected from multiple clinical sources which includes pathology data from the APLIS, tumor marker data from the Clinical Pathology Lab Information System (CPLIS), and clinical staging and treatment data from CRIS.

The challenges pertained to this process are identifying the same patient across various hospital sources and collecting the accurate data in precise context. These barriers are overcome by common linking patient identifiers and information and this helps in tracking the same patient information across multiple sources. This also reduces the chances of errors in data recording. The resource employs this solution to address the challenges that are largely due to a manual data collection and recording by the data manger. This helps in keeping the focus only on data quality. The next challenge is to collect data that requires to be monitored for context before it can be incorporated into the database. This is important for the data that has temporal relationship. The potential solutions to these challenges are automated data retrieval through electronic queries of existing systems for example electronic medical record (EMR) and cancer registry.

De-identification is the process which includes isolation of patient clinical identified data sets that are kept in the medical records from the research de-identified data sets. This process is done by honest brokers who act as a barricade between clinical identified data sets and totally de-identified research data sets. The honest broker collects clinical data, identifies the patient identifiers, separates them by assigning the research de-identified number and finally made de-identified data set available to research community. The NMVB resources assign each case a 4-digit de-identified case identification number and linkage to the patient health information is kept by the honest brokers. The NMVB database at no point in time present any linkages to the patient identifiers (medical record number, data of birth, social security number) to end users.

To facilitate the standardized clinical annotation process of biospecimens and to automate the process of annotation, the NMVB project team incorporated the NAACCR standard [19], CAP cancer protocol [20] and ADASP [21] guidelines for mesothelioma as a well recognized, significant and standardized sets of data elements in their respective domain. These standards include a series of reporting guidelines for diagnostic pathology reports and outcome related descriptors for the vast majority of human malignancies. Each CAP guideline is comprised of checklists with the data elements to describe the gross as well as microscopic attributes of the neoplasm including pathological staging and perifocal reactions such as margins, angio- as well as perineural invasion with specified valid values for each data element that are important for clinical decision-making and prognostication of individual cases.

The process of developing the CDEs for the NMVB has authenticated this initiative that can successfully direct to the implementation of strong human mesothelioma-related CDEs that assist in the collection of high quality data for the research community. The CDE are developed by NMVB CDE subcommittee that included experts from various clinical and research fields e.g. pathologists, thoracic surgeons, epidemiologists, bioinformaticians, biostatisticians, data managers, cancer registrars, and research scientists.

Finally the purpose was to create datasets that should provide value to the end users in future. Furthermore, the definitions of the CDEs and their associated descriptors need to be clearly understandable to all those who collects data. Through the use of ISO compliance and accepted data standards the goal of collecting annotation data of high quality was achievable. The only way in which information from multiple databases can truly be shared and made useful is through the careful use of unequivocal, well defined, consistent and structured metadata. Informaticians and database developers provided the structural link that brought the CDEs together in the database, addressed technical issues, and provided guidance related to implementation of the CDEs at local institutions.

### Presentation of NMVB Web Base Query Tool

The CAE model enables standards-based manual annotation of biospecimens with associated well-characterized clinical information. It supports importing structured data from various clinical information systems such as APLIS, CPLIS and cancer tumor registries allowing the integration of multimodal annotations within the cancer centers, providing a complete picture of a patient's disease [12]. Additionally, the CAE is based on such a model that aids in developing and conveying the semantic interoperability of the data system by describing the common data elements in the form of metadata or data descriptors (about the content, quality, condition, and other characteristics of the data) and by using controlled vocabulary and ontology, in order to make the data understandable and sharable for end-users and flexible for the system. Hence, the overall advantage of this suite over the legacy biospecimen annotation systems includes the shared responsibilities of individual institutions for services and implementation of the required standards and vocabulary that fosters data sharing effortlessly.

## Conclusion

The NMVB stands as a nation wide largest resource for mesothelioma archived and prospective biosepcimens with associated clinical, pathology, recurrence, follow-up and treatment data and act as a central resource for investigators using experimental methods in translational, histopathology, validation and outcomes measures. This resource is built upon a robust and efficient informatics architecture that facilitates well organized management, standardized collection and detailed clinical annotation of cases across multiple collaborative sites. This set-up provides a manual of operation, a histopathology guide, and a database with common data elements for characterization of mesothelioma biospecimens, multimodal datasets and a quality assurance and control process.

The NMVB web based query tool acts as a central source that provides a mechanism for researchers to efficiently search clinically annotated datasets and biospecimens that are pertinent to their research areas. The web-based query tool ensures patient health information protection by disclosing only de-identified data with Institutional Review Board (IRB) and scientific review committee approved standards and protocols. Additionally NMVB web site facilitates an online process of requesting mesothelioma biospecimen, acces statistical query tool for public view and access to approved investigator query tool to potential investigator. The biospecimens disbursement and database access to patient de-identified clinical data is granted after Research Evaluation Panel (REP) approval that includes experts on mesothelioma, biostatics and pathology. Different methods are adopted to market the resource that includes brochures, a website, and a booth that is being used to market the resource at scientific meetings. In fact NMVB presents as a fundamental platform for the development and implementation of an integrated tissue banking program and facilitates other tissue banking efforts through the members of the collaborative resource and their associated publications.

## Abbreviations

ADASP:  Association of Directors of Anatomic and Surgical Pathology; AJCC:  American Joint Committee on Cancer; APLIS:  Anatomic Pathology Lab Information System; caBIG:  cancer Bioinformatics Grid; caDSR:  cancer Data Standards Repository; CAE:  Clinical Annotation Engine; CRIS: Cancer Registry Information System; CAP: College of American Pathologist; CBCTR: Cooperative Breast Cancer Tissue Resource; CDC:  Center for Disease Control and Prevention; CDE:  Common data element; CPCTR: Cooperative Prostate Cancer Tissue Resource; CPLIS: Clinical Pathology Lab Information System; EA:  Enterprise Architect; EDRN:  Early Detection Research Network; EMR:  Electronic Medical Record; HIPAA: Health Insurance Portability and Accountability Act; IATA: International Air Transport Association; IRB: Institutional Review Board; ISO:  International Organization for Standardization; MARF: Mesothelioma Allied Research Foundation; NAACR:  North American Association of Central Cancer Registries; NCI: National Cancer Institutes; NIOSH: National Institutes of Occupational health and Safety; NMVB: National Mesothelioma Virtual bank; NYU: New York University; PCABC: Pennsylvania Cancer Alliance Bioinformatics Consortium; PHI:  Protected Health Information; QA: Quality assurance; QC: Quality control; REP:  Research Evaluation Panel; SPORE:  Specialized Programs of Research Excellence; TBIS: Tissue Bank Information System; TMA: Tissue microarray; TNM: Tumor/Node/Metastasis; UML: Unified Medical Language; UPENN: University of Pennsylvania; UPITT: University of Pittsburgh;  UPMC: University of Pittsburgh Medical Center.

## Competing interests

The authors declare that they have no competing interests.

## Authors' contributions

MJB, WA, SKM, LS, GF, JM and AVP contributed equally to the first draft of this manuscript. MJB, the chair for the Coordinating Committee, is responsible for leading the efforts of developing the requirements for the central database. MJB, WA, SKM, AVP, RD, JM, MF and HIP have contributed in study design, implementation and quality assurance of the database and tool. LS and AKP have contributed in the development and implementation of software tools for the data annotation and query engine in the web based interface, and incorporation of other existing standards. SBW, AS and GF have played an important role in implementation of Cancer registry data standards into the database and collection and quality assurance of follow-up and epidemiological data. NBW is the overall Project coordinator for this project. All authors have reviewed and commented on successive drafts of the manuscript and have provided the first author with approval of the final manuscript.

## Pre-publication history

The pre-publication history for this paper can be accessed here:



## Supplementary Material

Additional File 1 Honest Broker services for Tissue Banks and Clinical data. Click here for file
