# Supplementary material for: National Mesothelioma Virtual Bank: A standard based biospecimen and clinical data resource to enhance translational research
Source: BMC Cancer. 2008 Aug 13;8:236. doi: 10.1186/1471-2407-8-236 (PMC2533341; doi:10.1186/1471-2407-8-236)
Supplement: Additional File 1 — Honest Broker services for Tissue Banks and Clinical data. [file 1471-2407-8-236-S1.docx]

**A multi-disciplinary approach to honest broker services for tissue banks and clinical data: a pragmatic and practical model**

Dhir, R^1^, Grzybicki, D^2^, Bisceglia, M^1^, Winters, S^2^, Aamodt, R^4^, Swanson, D^3^, Becich, MJ^2^

^1^Dept. of Pathology, University of Pittsburgh, Pittsburgh, PA, United States

^2^Dept. of Biomedical Informatics, University of Pittsburgh, Pittsburgh, PA, United States. ^3^Dept. of Pharmacology, University of Pittsburgh, Pittsburgh, PA, United States. ^4^Aamodt Enterprises, Gaithersburg, MD, United States.

**Submitting author:**

Rajiv Dhir, M.D.

Associate Professor of Pathology, University of Pittsburgh Director, Health Sciences Tissue Bank, University of Pittsburgh UPMC Shadyside-Presbyterian Hospital

5230 Center Avenue, Room WG 07

Pittsburgh PA 15232

E [mail: dhirr@msx.upmc.edu](mailto:dhirr@msx.upmc.edu)

Phone: (412) 623-1321.

Fax: (412) 682-6450.

**Abstract**

**Background:** Honest broker services are an essential component of research support provided by tissue banks. The honest broker provides a firewall between clinical and research activities. Clinical information so critical to phenotyping biospecimens is stripped of personal health identifiers identified by HIPAA. Research material (specimens) may be linked to the patient information by a code, but the honest broker ensures that researchers cannot identify patients. Likewise the honest broker ensures that research data, which is generally not clinically validated, is not used for clinical care. Current research requirements generally include biological specimens with significant clinical data annotation. The complexity of these processes raises a variety of issues and concerns regarding the precise role of the honest broker and their interaction with data. There also is an obvious need for software solutions to make the task of de-identification easier.

**Methods:** The University of Pittsburgh has implemented a novel IRB approved mechanism to address honest broker functions to meet the biological specimen and data needs of researchers. The Tissue Bank partnered with the Cancer Registry, the Clinical Outcomes group, Medical Oncology, Radiation Oncology, Pathology and Oncology Informatics. This consortium submitted a joint IRB proposal to create an honest broker facility. The Tissue Bank stores biological specimens. The Cancer Registry culls data and annotating information, as part of state- and federal-mandated functions and collects clinical progression, treatment and outcomes of cancer patients. In addition, the Cancer

Registry also collects data elements specifically for research purposes (approved via a separate IRB submission). The clinical outcomes group is involved in patient safety and health services research. Radiation Oncology and Medical Oncology provide critical treatment related information, with this information present as electronic files or available for abstraction from paper records. Pathology and Oncology Informatics have designed software tools and assist in designing mechanisms for querying for availability of biological specimens, extracting data, and de-identifying biological specimens and annotating data, which serve the needs of clinical and translational researchers. The employees of this conglomerate have honest broker agreements with the University of Pittsburgh and the Medical Center. This provides a large task force for honest broker activities, especially since best practices dictate that an honest broker should not participate in the analysis or aggregation of research data from the protocol that supports their work.

**Results:** The honest broker system has been an IRB approved institutional entity at the University of Pittsburgh since 2003. The honest broker system currently includes 33 certified honest brokers encompassing the multiple partners of this system. The honest broker system has handled over 300 requests over the past 4 years, with a 25% increase in volume each year.

**Conclusions:** This collaborative honest broker model has proven to be robust and provides a highly functional solution to these critical clinical and translational research activities.

**Background**

The last decade has seen significant advances in molecular biology (genomics and proteomics) and translational research. These new initiatives have resulted in a growing demand for specific, highly annotated human tissues and other biological specimens [1- 3]. This growing demand has reinforced the importance of tissue banks as a major part of the necessary infrastructure of any institution/ research initiative seeking to address biologically and clinically relevant issues. In addition, many of these research initiatives require extensive annotating information that is not present in one data source.

Two areas particularly need annotating information. These are tissue-based research and health services based research, which requires patient information for research assessment. Health services research includes outcomes focused research, assessing impact of different therapeutic regimens, research focused on quality and safety of health care, research to evaluate quality assurance, quality control and errors, as well as research focused on impact of different information system on overall quality of health delivery, patient education and error reduction.

The past few years have also seen a significant structured movement to protect the confidentiality of research study participants. Although the concept of an honest broker has been around for more than a decade, the advent of the Health and Insurance Portability and Accountability Act (HIPAA) [4] further emphasized the need for systems/ mechanisms to identify and remove personal health identifiers (PHI) from research

information. There are facility/institution-specific regulations mandating policies on patient confidentiality. The Institutional Review Board (IRB) also provides input and direction regarding policies and procedures impacting access to patient information. Finally institutions are also cognizant of prevailing views regarding legal and ethical issues. It is important to develop protocols to protect patient identifiers and confidentiality in the current environment.

The need for data as well as the need for subject confidentiality protection resulted in a log jam blocking data aggregation and disbursement. This conflict exposed the lack of preparedness of major institutions to collect, collate and disburse data elements needed for projects while maintaining patient privacy. The result was that access to well documented tissue specimens, using normalized descriptors, became an important impediment to the progress of research projects [5].

The Cancer Registry and the Health Sciences Tissue Bank engaged in discussions to evaluate mechanisms for addressing this issue. The major players in the research field were identified. The Health Sciences Tissue Bank, the Pathology Laboratory Information System, the Cancer registry, the Clinical Research Informatics Service, the Clinical Outcomes group, Radiation and Medical Oncology, Pathology and Oncology Informatics and the “Electronic Medical Record” team were considered key players. This list might not encompass every possible entity that could play a role; nonetheless it captures the major players involved in the aggregation and provision of specimens and data. Policies and procedures were established to serve as guiding principles.

**Methods**

The requests for biological specimens and data for research purposes have increased significantly over the years. Data requests have become increasingly complex. This increased complexity is partly related to outcomes related initiatives to evaluate biomarkers and their role in guiding therapy or predicting outcome. In addition, awareness of confidentiality issues has increased significantly since the implementation of HIPAA.

The primary request for research projects consist primarily of:

1. Tissue and biological specimens only.
2. Clinical (phenotype) data, most frequently pathology data.
3. Outcomes information including treatment, progression and vital status We evaluated tissue and data requests at the University of Pittsburgh and found that 20% of research projects needed biological specimens only, 10% of research projects needed outcomes information, while the remaining 70% needed more fully annotated tissues requiring phenotypic (clinical) data. The annotation varied from easily accessible (e.g. pathology data) to complex (pre-therapy and post-therapy information). This suggested the need to design a system that could provide research biological specimens annotated with patient data while protecting the confidentiality of patient information, while fully meeting the requirements of federal regulations [6]. This required implementation of a system that is HIPAA compliant and provides human subjects protection. The resulting system for this process was based on the Honest Broker Concept.

**Human Subjects Protection – The Honest Broker Concept**:

The tissue/databank ensures protection of patient identity through "The Honest Broker Concept." The honest broker is an individual/organization/system which acts on, or on the behalf of, the tissue/databank. The role of the honest broker is to collect and provide health information to research investigators in such a manner whereby it would not be reasonably possible for the investigators, or other individuals, to identify the subjects directly or indirectly. The “honest broker” or “tissue/data bank trustee” acts as a well defined barrier between the clinical environment (in which fully identified confidential patient information is routinely exchanged as part of medical care) and the general research community (in which all information must be completely de-identified). The honest broker also ensures that research data, which is generally not clinically validated, is not used for clinical care [7].

In our rendition, **the honest broker is not part of either the clinical or research team**. This is important to ensure confidentiality and honest research. The honest broker is the only entity that can link research identifiers and clinical identifiers. This transfers control and responsibility of the de-identification process to an independent third party, the honest broker, thereby reducing the risk of conflict of interest. Personal and clinical identifiers (names, addresses, medical record numbers etc.) are limited to the clinical space. The research identifiers (i.e. “subject 12432”) cannot be traced back to the personal or clinical identifies except through the honest broker’s linkage codes.

**This concept differs from anonymization.** Anonymization is a one-way process in which the linkage between personal identifiers and research identifiers is removed.

Anonymization precludes any subsequent updating of data. The process of data annotation with the particular specimen stops when anonymization is performed. The process of having the honest broker assign linkage codes (re-identification codes) allows information to be updated at anytime in the future. The honest broker can identify the patient by means of the linkage code, access information related to this patient from the clinical domain, and provide updated information to the researchers in a deidentified fashion, using the original linkage code. The link between codes must be retained and protected by the honest broker. Subsequent requests to update information on research protocol participants (research cohort) must be conducted through the honest broker. The honest broker system is therefore an upgrade to the process of anonymization. Anonymization essentially provides information up tot the time of accrual, whereas the honest broker concept allows information to be updated in a manner that is consistent with current legal and ethical protocols.

Discussions involving the Cancer Registry and the Health Sciences Tissue Bank identified the major sources of tissue and biological specimens and annotating data for research use. The privacy rule of the HIPAA of 1996 permits access to protected health information without patient authorization in a limited number of situations [4]. One frequent situation is where the protected health information is being used in a deidentified fashion. The honest broker plays a prominent role in this scenario, since neither the federal policy nor HIPAA regulations require prior written consent or authorization of patients when using existing health information in a de-identified fashion. The honest broker can be a part of the facility providing the data. In addition the honest broker can

be a business associate of the facility. This approach allowed us to expand the circle of participating facilities. We decided to include division/departments involved in data aggregation as well as facilities that were creating and implementing software solutions and tools for these groups as participants for this initiative. The software groups included Pathology and Oncology Informatics and the Electronic Medical Records team. This list may not include every possible entity that could play a role; nonetheless it does capture the major players involved in aggregation and provision of specimens and data, and designing software tools for these efforts.

The facilities currently part of the “Honest broker facility” and their role in this initiative is described below.

**Participating facilities**:

1. **The Health Sciences Tissue Bank**: The Health Sciences Tissue Bank is the main institutional infrastructure for collecting tissue and other biological materials for research. These research specimens are stored in a de-identified fashion, annotated with linkage codes, because of confidentiality issues. However the linkage codes allow access to specific information regarding the donor. This is important since many research projects require not only tissue and biological specimens but also additional data regarding family history, treatment history, and outcomes.
2. **The Pathology Laboratory Information System**: This is the clinical system used for reporting pathology information. This repository contains extensive

information regarding clinical evaluation of tissue and other biological specimens. This information is extremely useful to provide a better understanding of the composition of the research specimen. The system stores clinically reported information pertaining to tissue specimens (biopsy and resection reports), cytology specimens (exfoliated as well as aspirate specimens), and other biologic specimens (blood/blood products/urine/other biological specimens).

1. **The Cancer Registry**: The Registry performs the state-mandated function of collecting information on cancer patients. The information collected pertains to both diagnostic details as well as follow up information. The data collected by the Registry consists of a set of defined data elements that are part of a standardized set of common data elements. We have further modified this approach by adding additional data elements, of primarily research value, as part of a separate IRB approved initiative.
2. **The Clinical Outcomes group**: This institutional entity collects and provides information pertaining to ongoing clinical trials, health services research and patient safety research.
3. **Radiation and Medical Oncology**: Radiation and medical oncology are important caregivers for oncologic diseases. The clinical database of these two entities provides critical information regarding therapeutic intervention and responses to those specific therapies. Information accrued from Radiation and Medical Oncology is therefore critical in providing insight regarding patient response to therapeutic protocols.
4. **Pathology and Oncology Informatics**: This growth is responsible for designing and maintaining the informatics infrastructure for collection, storage and disbursement of annotating information. It is important to affiliate this group with the honest broker infrastructural development since Pathology and Oncology Informatics designs, tests and maintains the tools needed for the other components of the honest broker system. Some of these include software packages needed for Inventory Management by the Health Sciences Tissue Bank, data aggregation software packages for the Cancer Registry and clinical outcomes group, clinical information and research information recording mechanisms for Medical and Radiation Oncology, and de-identification software packages needed by many participating facilities (Health Sciences Tissue Bank, Cancer Registry, the Electronic Medical Record team and others). NOTE: Our Pathology and Oncology Informatics groups were recently merged into the new Department of Biomedical Informatics as of June 2006, see <http://www.dbmi.pitt.edu>).
5. **The University of Pittsburgh Health Systems Information Services Division**: Most clinical data is captured in an electronic form in various hospital information systems. This includes patient history, details of surgical and radiological procedures, therapeutic interventions and follow-up information. The clinical component of the electronic medical records consists of information in an identified form. However the transfer of this information into the research domain requires de-identification of this information. The electronic medical record team therefore serves as a gatekeeper for this information and oversees implementation of appropriate de-identification protocols prior to the

incorporation of this data into research databases. The electronic medical record team also plays a critical role in performing queries for specific research requests. This activity helps identify appropriate patient populations for research projects. These identified patient lists then need to undergo de-identification.

In this concept at least one individual is acting as an honest broker at each of the facilities listed above. For clinical and translational research studies in oncology, the Cancer registrars are extremely valuable since their federal mandate and the job specifications allow them ready access to clinical information on cancer patients. In addition, they are not involved in specimen banking or research and thus do not have access to the data annotating tissue bank samples or the results of the research studies. The inclusion of the cancer registry into an honest broker system facilitates data accrual from this purely clinical data entity which maintains updated information on all oncology patients. This updating is done every six months and is part of the state-mandated function of the cancer registry.

The “Institutional Honest Broker” system ensures that the honest broker ("trustee") is the only person who can link a patient with the tissue bank number that identifies that patient. The Institutional Honest Broker system also provides a process via which new clinical outcome information can be added to a file identified only by a code number, rather than a name. This creates a fail-safe mechanism for communicating with patients in the extremely rare event of an IRB directed dissemination of important research data to the patient or their survivors.

It was decided to incorporate the above named groups, involved in tissue and data aggregation with possible research application, into an Institutional Honest Broker system.

The University of Pittsburgh Academic Health Center consists of two closely interacting, but legally separate, entities. These are the University of Pittsburgh, which oversees primarily the research activities, and the University of Pittsburgh Medical Center (UPMC), which oversees clinical activity and in which the clinical data resides. Potential legal/ ethical issues pertaining to the creation of this system were discussed with the Institutional Review Board (IRB) of the University of Pittsburgh as well the legal team of the UPMC. A formal IRB application for this “Honest Broker Facility” incorporating the comments and suggestions of the IRB and the legal team of the University of Pittsburgh Medical Center Health Systems was approved by the IRB and formally went into effect in May 8, 2003.

The employees of the Honest Broker Facility have honest broker agreements with the University of Pittsburgh and the University of Pittsburgh Health Systems. This Honest Broker Facility encompasses several separate departments and divisions. Each of these entities has contributed by providing personnel into the honest broker pool. This arrangement has provided a large task force for honest broker activities, which is important since an honest broker should not be involved with the research requiring honest broker services. This approach ensures lack of conflict for the individual engaged in honest broker activities, thereby creating an appropriate work environment.

**Honest Broker Process**: The honest broker certification process requires completion of IRB mandated education modules. These modules are Research Integrity, Human Subjects Research in Biomedical Sciences, and HIPAA Researchers Privacy Requirements. The education modules can be completed via the Web at the University of Pittsburgh IRB web site (<https://cme.hs.pitt.edu/)>. A certificate of completion is generated once each module has been completed. In addition the honest broker also has to enter into a business associate agreement (see supplemental file – NEED MORE SPECIFIC REFERENCE). An individual can become a certified honest broker, once these administrative requirements have been completed.

The honest broker facility provides an update to the IRB every six months. The update is in opportunity to add/delete honest brokers. The Institutional Honest Broker system at the University of Pittsburgh has assigned overall administrative responsibility for the honest broker service to the Manager of the Cancer Registry. However this oversight can be provided by the leaders of any of the participating entities.

The Pathology and Oncology Informatics division has designed an Honest broker Data Request Tracking Tool for the honest broker system. This tool provides the interface for entering descriptive detail information pertaining to a research project requiring honest broker services. This tracking tool is password protected and is located within the firewall of the University of Pittsburgh. After logging into the system, a menu of options is available to the honest broker. The honest broker handling a particular request enters all the information about the research project into the database using the initial data-entry

screen of this tool. The initial data-entry screen captures information pertaining to the investigator, the nature of the request, as well as important workflow issues like requested turnaround time, IRB status and approval number. In addition this screen also captures information pertaining to billing, in case the services provided will be compensated through an institutional account, rather than grant funded mechanisms. This tool has a built-in query capability. The honest broker designates the fields required for the data sources, the disease category, method of output for tissue/ biological specimens and data, the method of distribution and the purpose of the request. The honest broker alerts their supervisor once all project information has been entered into the tracking tool. The supervisor reviews project details and provides input and approval. This tracking tool is used to follow a research tissue/data request from start to finish. This provides information regarding turnaround time as well as time spent on a project. All of this information is summarized and available in the final "complete request" snapshot of the tool.

**De-identification protocols**: The de-identification of patient samples and data is performed using a variety of tools. The Pathology Lab Information System, CoPath, has limited de-identification capabilities. The electronic medical record system also has de- identification software systems. The honest broker system can be utilized for de- identifying specimens/data. The honest broker retains the codes for the specimen/data provided. In addition the Clinical Research Informatics Service (<http://www.dbmi.pitt.edu/cris/> ) in the Department of Biomedical Informatics has created a HIPAA compliant de-identification engine. This de-identification engine has

been certified by the IRB of the University of Pittsburgh as well as by the University of Pittsburgh medical Center security office for generating de-identified output from a variety of free text medical reports. This engine identifies all HIPAA mandated PHI, eg. names and replaces them with a de-identified tag and replacement letters. If the same person is encountered in multiple places in the same report, the same replacement letters are used for every occurrence. Similarly dates are replaced by an offset which allows intervals among aggregated reports to still allow for interval determination. An example of a deidentified report generated by this engine is shown in figure 5. The system generates a linkage file for each patient. This file is stored on a secure server.

**Data sources**: The collaborative honest broker service utilizes multiple sources of data. These include clinical applications (Pathology Laboratory Information Services, Radiation Oncology Systems, Outpatient Systems and Hospital Information Systems), Clinical Trials related applications, Cancer Registry applications, and Tissue Banking Inventory and Information Systems. In addition paper-based records in physician offices and legacy records in the hospital may be used.

**Results:**

The honest broker facility received IRB approval in May, 2003. Four months were required to train the personnel and take care of the paperwork for certification of the honest brokers. The existence of the system was then announced to the staff and faculty of the University of Pittsburgh in October 2003.

The initial response to the facility was initially slow and the last three months of 2003 generated only six requests for the honest broker facility. The volume of research requests increased significantly in 2004. The calendar year 2004 generated 148 requests. The calendar year 2005 generated 449 requests. The first eight months of 2006 had already generated 398 requests. Handling of requests in the second and third quarters of 2006 has been hampered by having enough staff to fulfill all requests.

The requests for the honest broker facility have come from all major oncology areas. The honest broker facility has handled requests from all the major organ type groups. These include the pulmonary group, the head and neck group, the gastrointestinal diseases group, the genitourinary and prostate group, the hematology group, the skin and melanoma group and the gynecology diseases group, including the breast group. It should be noted the Breast and Gynecologic Oncology Group has started using the facility starting January 2006.

The honest broker facility has received work requests for a variety of different tasks.
These include preparatory for research, research projects, presentations and abstracts,

quality and process improvement, assessment of incidence of disease, marketing of clinical program, as well as for patient safety initiatives, clinical quality control and quality improvement. We evaluated these requests to assess distribution by organ type.

**How does an investigator use the honest broker facility?** A researcher can approach any of the constituents of the honest broker facility with a research request. The research request can be for tissue, biological specimen, or clinical data. This specific component of the honest broker facility approached by the investigator evaluates the research requests and identifies the different components of the honest broker facility that would play a role in fulfilling the request. One of the constituent facilities is designated as the primary handler of the requests. This facility interacts with the other components involved in the request. This primary facility communicates with the researcher, ensures that all the requested tissue/biological specimens have been retrieved and collates the data. The entire set of tissue and biological specimens and annotating data is de- identified and then provided to the investigator.

**Accrual to clinical trials:** The honest broker system can also be used to assist in increasing accrual for clinical trials. The honest brokers can be used to identify patients eligible for recruitment into clinical trials, using defined search criteria. The clinical trial investigators would be provided de-identified listing of health information of potential eligible subjects. The clinic trial investigators could then make eligibility decisions based on criteria for the trial. The honest broker can then contact the patient's personal physicians and make them aware of which of their patients are eligible for a specific

clinical trial. The primary physicians should then talk to their patients about the clinical trial and ascertain their interest in participation in that trial. The patients can contact the investigators directly. Alternatively they can provide written authorization to their physicians, allowing the physicians to contact the investigators. **Direct contact of patients by the honest broker ("cold-calling") is prohibited by IRE regulations**.

**Use by repositories other than the University of Pittsburgh:** This honest broker system has been used by entities other than the University of Pittsburgh. A similar model has been applied by the Cooperative Prostate Cancer Tissue Resource [3, 8, 9] as well as the Pennsylvania Cancer Alliance Bioinformatics Consortium. In addition, similar protocols were adopted for case retrieval for the Shared Pathology Informatics Network (SPIN) validation studies [10].

**Discussion:**

The honest broker facility is now a well established mechanism for de-identified tissue and data disbursement. This facility has become very popular in a short period of time. This is borne by the incremental increase in the use of the facility over the last four years. The popularity of the honest broker facility has started creating logistical issues, especially pertaining to staffing and turnaround issues.

There are certain aspects of the honest broker facility that need to be considered when creating a facility similar to the one at the University of Pittsburgh.

**Training of honest brokers**: This is an important aspect of maintaining uniform functionality of the honest broker facility. The facility has seen a significant increase in honest brokers on the last four years. The honest broker facility started with five honest brokers. The facility now has 33 honest brokers. The initial aspect of training focuses on explaining the compliance guidelines and objectives of the honest broker facility, discussing the philosophy of existence of the facility and completion of the IRB mandated research models. These steps provide the new honest broker is with conceptual details of the honest broker facility. The honest brokers are then trained on the software available for extracting data. This includes the honest broker tracking tool as well as mechanisms for de-identification.

**Specialization of cancer registrars**: Another important parallel initiative has focused on
creating a pool of specialized cancer registrars. These cancer registrars work in a specific

organ system of cancer program. They are involved in collecting information on patients with a specific cancer. The information collected consists of the state-mandated reporting requirements from the Cancer Registry. In addition, these "specialized" cancer registrars collect additional data elements for research purposes that have been approved by the IRB of the University of Pittsburgh. These cancer registrars also frequently approach the clinical caregivers to resolve data discrepancies among different sources.

These cancer registrars therefore focus in on a particular organ system of cancer program. Their work could be considered representative; however this specialized approach serves to increase their knowledge base and awareness of issues related to a particular subset of tissues and tumor types. These registrars perform data entry for the state-mandated clinical function of the Cancer Registry. In addition they handle specific requests for their area of concentration. This ensures a higher quality of data entry and retrieval.

In addition to increasing the clinical and translational research skills of the specialized cancer registrars, they become experts in a variety of clinical information systems from which they extract phenotypic data. They also develop a variety of informatics skills in the areas of data processing, data de-identification and the use data warehouses. They have particularly developed skills in data mining tools (both commercial as well as developing their own customized algorithms for clinical and translational research).

**Increased availability of Tissue/Data to investigators**:

Numerous annotated tissue repositories already exist in this institutions and its affiliated cancer center. These include frozen as well as paraffin embedded tissue materials and other biological materials. The overall goal is to make them available to a wider research community, in a manner that is efficient, rapid, and compliant with legal and ethical concerns. There is significant awareness locally about the benefits of expanding utilization of our resources in collaborative projects. The creation of an institutional tissue resource as well as an institutional honest broker facility has served to accelerate access to tissue, biological materials and annotating data. Furthermore, many tissue bank- focused projects do not take into account the vast resources of paraffin archives, housed in many academic pathology departments [2, 6], that are available for use. This initiative will serve to bring down barriers at the institutional level and provide access to all forms of biological materials and data.

**Funding:** Initial provision of adequate resources is required to ensure the success of this institutional facility. There has been upfront investment by the institution in terms of personnel. The honest broker facility also has been incorporated in grant submissions to provide committed funding for these activities. The honest broker facility is consulted by the principal investigator submitting the grand proposal. The broad outline of the project is discussed. An estimate is made of the amount of time needed to fulfill projected needs of the project. The principal investigator then incorporates the anticipated personnel requirements in the budget of the proposal. In addition this facility also functions on a

fee-for-service basis. The fee-for-service mechanism applies to work done on non-grant funded initiatives. The fee-for-service is based on an hourly rate for providing honest broker/de-identification services, data accrual, creation of database, and chart review. These different monetary mechanisms have helped provide resources for the facility to survive and grow.

**Conclusions:**

The creation of an institutional honest broker facility has created a robust mechanism for data accrual and disbursement. In addition it has led to the development of a significant informatics infrastructure to support this facility's functions. This has decreased turnaround time for providing data associated with samples provided to investigators. It is hoped that this system will promote more robust, efficient and clinically and biologically relevant studies of biomarkers. Studies resulting from the creation of this facility may allow for better classification of cancer types, more accurate assessment of disease prognosis, a better ability to identify the most appropriate individuals for clinical trial participation, and better surrogate markers of disease progression and/or response to therapy. In addition, the biomedical informatics infrastructure and the honest broker tools created to serve the honest broker facility will be made available for use by outside institutions. It is hoped that this approach focused on sharing our experience and software tools will benefit research on a more global scale.

**Acknowledgements:**

We acknowledge the following contributors to the development of the honest broker facility:

*University of Pittsburgh and the University of Pittsburgh Cancer Institute*:

George Michalopoulos, Ronald Herberman, Christopher Ryan, Stephen Raab, Harpreet Singh, and Theresa Colecchia.

**References:**

1. Becich MJ: The role of the pathologist as tissue refiner and data miner: the impact of functional genomics on the modern pathology laboratory and the critical roles of pathology informatics and bioinformatics. Mol Diagn 2000, 5(4):287-299.
2. Eiseman E, Rand Corporation.: Case studies of existing human tissue repositories: "best practices" for a biospecimen resource for the genomic and proteomic era (<http://www.rand.org/publications/MG/MG120/)>. Santa Monica, CA: RAND; 2003.
3. Patel AA, Gilbertson JR Parwani AV Dhir R Datta MW Gupta R Berman JJ Melamed J Kajdacsy-Balla A Orenstein J Becich MJ Cooperative Prostate Cancer Tissue Resource An informatics model for tissue banks--lessons learned from the Cooperative Prostate Cancer Tissue Resource. BMC Cancer. 2006 May 5;6:120
4. Health Insurance Portability and Accountability Act of 1996 [<http://aspe.hhs.gov/admnsimp/pl> 104191 .htm]
5. Gilbertson JR, Gupta R, Nie Y, Patel AA, Becich MJ: Automated clinical annotation of tissue bank specimens. Medinfo 2004, 1 1(Pt 1):607-610.
6. Department of Health and Human Services. 45 CFR (Code of Federal Regulations), 164.5 14(6)(2)(i). Standards for Privacy of Individually Identifiable Health Information (final) [[http://www.hhs.gov/ocr/regtext.html]](http://www.hhs.gov/ocr/regtext.html%5d)
7. Mertz JF, Sankar P, Taube SE and LiVolsi V. Use of Human Tissue in Research: Clarifying Clinician and Researcher Roles and Information Flow. J Invest. Med. 45:252-257. 1997.)
8. Patel AA, Kajdacsy-Balla A, Berman JJ, Bosland M, Datta MW, Dhir R, Gilbertson J, Melamed J, Orenstein J, Tai KF et al: The development of common data elements for a multi-institute prostate cancer tissue bank: the Cooperative Prostate Cancer Tissue Resource (CPCTR) experience. BMC Cancer 2005, 5:108.
9. Melamed J, Datta MW, Becich MJ, Orenstein JM, Dhir R, Silver S, Fidelia­Lambert M, Kadjacsy-Balla A, Macias V, Patel A et al: The cooperative prostate cancer tissue resource: a specimen and data resource for cancer researchers. Clin Cancer Res 2004, 10(14) :4614-4621.
10. Patel AA, Gupta D, Seligson D, Hattab EM, Balis UJ, Ulbright TM, Kohane IS, Berman JJ, Gilbertson JR, Dry S, Schirripa O, Yu H, Becich MJ, Parwani AV; Shared Pathology Informatics Network. Availability and quality of paraffin blocks identified in pathology archives: a multi-institutional study by the Shared Pathology Informatics Network (SPIN).

BMC Cancer. 2007 Feb 28;7:37.
